# Evaluating initial usability of a hand augmentation device across a large and diverse sample

**DOI:** 10.1126/scirobotics.adk5183

**Published:** 2024-05-29

**Authors:** Dani Clode, Lucy Dowdall, Edmund da Silva, Klara Selen, Dorothy Cowie, Giulia Dominijanni, Tamar R. Makin

**Affiliations:** 1 MRC Cognition and Brain Sciences Unit, University of Cambridge, Cambridge, UK; 2 Institute of Cognitive Neuroscience, University College London, London, UK; 3 Dani Clode Design, Cambridge, UK; 4 Department of Psychology, Durham University, Durham, UK; 5 Bertarelli Foundation Chair in Translational Neural Engineering, Neuro-X Institute, Ecole Polytechnique Fédérale de Lausanne, Lausanne, Switzerland

## Abstract

The advancement of motor augmentation and the broader domain of human-machine interaction relies upon a seamless integration with users’ physical and cognitive capabilities. These considerations may dramatically fluctuate between individuals, based on their age, form and abilities. There is a need to develop a standard for considering these diversity needs and preferences to guide technological development, and large-scale testing can provide us with evidence for such considerations. Public engagement events provide an important opportunity to build a bi-directional discourse with potential users for the co-development of inclusive and accessible technologies. We exhibited the Third Thumb (Dani Clode Design) at a public engagement event and tested participants from the general public, who are often not involved in such early technological development of wearable robotic technology. We focused on wearability (fit and control), ability to successfully operate the device, and ability-levels across diversity factors relevant for physical technologies (gender, handedness, and age). Our inclusive design was successful in 99.3% of our diverse sample of 596 individuals tested (age range 3-96 years). Ninety-eight percent of participants were further able to successfully manipulate objects using the extra Thumb during the first minute of use, with no significant influences of gender, handedness, or affinity for hobbies involving the hands. Performance was generally poorer among younger children (aged ≤ 11 years). Although older and younger adults performed the task comparably, we identified age costs within the older adults bracket. Our findings offer tangible demonstration of the initial usability of the Third Thumb for a broad demographic.

## Introduction

Technology is changing our very definition of what it means to be human. Machines are increasingly becoming a part of our everyday lives, and even our minds and bodies. An exciting area for future technology is motor augmentation, which is designed to enhance or extend the physical capabilities of humans (1–3). This can include wearable devices such as exoskeletons (4) or extra robotic body parts (5, 6), as well as teleoperation of remote devices (7). Although serving different functions and driven by distinct technological breakthroughs, these devices are all designed to advance motor capabilities beyond current biological limitations. For example, having an extra robotic thumb could increase your range of movement and improve your hand grip strength, precision, and dexterity. Such extended abilities may allow the user to perform tasks that might be otherwise challenging or impossible to complete with one hand; or enable them to perform complex multi-handed tasks without having to coordinate with other people. Likewise, exoskeletons can allow workers to lift and carry heavy loads without risk of injury or can provide extra strength to the wearer to reduce strain on the body. For these reasons, wearable motor augmentation technologies have the potential to improve productivity and safety in a variety of industries, such as construction, manufacturing, and more (8, 9). Beyond improving the quality of life for healthy individuals who want to enhance their productivity, the same technologies can also provide people with disabilities new ways to interact with their environment (10). For their versatility of purpose, future augmentation technologies could have an extensive influence on society and be beneficial to many people.

This increased intimacy between humans and technology opens up exciting new opportunities to improve society. However, as technology’s influence grows, it is our responsibility to ensure that everyone will have the opportunity to participate and benefit from these exciting technological advances to best achieve the desired societal consequences. Therefore, it is important that inclusion is taken into consideration during the earliest possible stages of the research and development process.

Including diverse populations has been a challenging topic to address within technology, particularly in software development and artificial intelligence (11). One example of technological failure due to the lack of inclusive design considerations, are automated speech recognition (ASR) systems converting spoken language to text, developed by companies such as Google, Apple, Amazon and Microsoft. These systems are integrated into consumer facing products like virtual assistants - Apple’s Siri and Amazon’s Alexa, as well as automated helplines and GPS navigation systems. Studies have found that these kinds of speech recognition technologies have various inequal biases, for example racial disparities in recognition, with an error rate favouring white speakers over black speakers (12); these technologies are also more likely to misunderstand or fail to respond to commands from users with accented English speech (13). Another example is the design of augmented reality (AR) technology, which overlays digital information on to the real world; some AR technologies have been found to be less effective for users with darker skin tones (14). In these cases, a lack of diversity and inclusion in the development and testing phases of these technologies resulted in negative consequences for some users.

In addition to these highly common and very broad issues that are being highlighted in the software-based domain, when building technologies requiring physical interactions with the human body and mind, other considerations become crucial. For example, women face a higher health risk from car accidents, due to car seats and seatbelts being primarily designed to accommodate ‘average’ male-sized dummies during crash testing (15). In addition, this one-size-fits-all approach disregards teenagers, disabled individuals, and the elderly. Another common example relates to the design of hazardous power and industrial tools, which were designed for a right-hand dominant use or grip, consequently causing more accidents when operated with the non-dominant hand of left-handers (16). Therefore, when building human-centred physical interfaces, and wearable technologies in particular, the burden of diversity and inclusion consideration is substantial. We argue that for technology to thrive, developers must anticipate its broad range of consumers. This is particularly challenging for futuristic technologies, where the specific use scenarios are still underdetermined.

**Figure 1:**
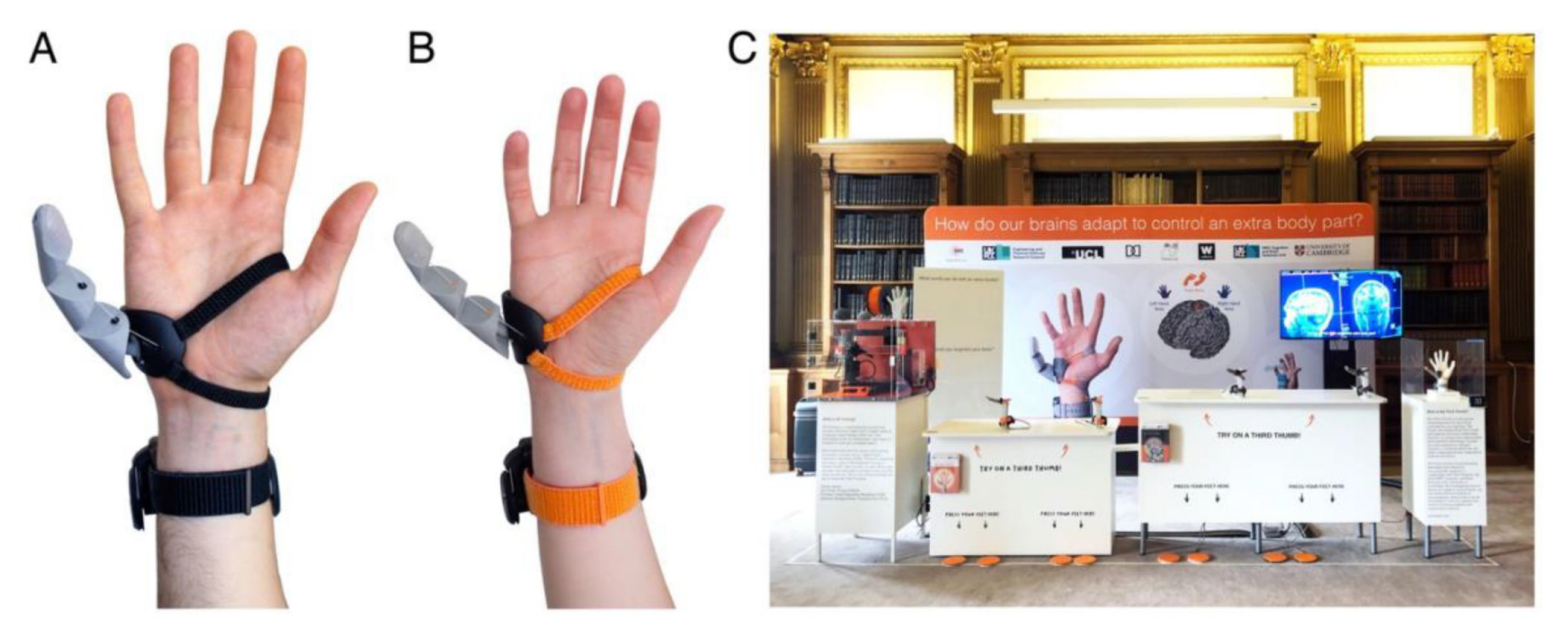
The Third Thumb exhibition setup. **(A)** The Third Thumb designed by Dani Clode Design, Large size. **(B)** Small size of the Third Thumb, predominately used by children. **(C)** Photograph of the final exhibition layout at the Royal Society in London.

The Third Thumb (Dani Clode Design; (17); Figure 1A) is a 3D-printed hand augmentation device that was designed to extend the motor abilities of an already fully functional hand. The Third Thumb was designed with user diversity in mind. The design was based on extensive initial testing by the designer in various previous public engagement activities, in combination with a more rigorous testing setting (17). So far, opportunities for empirically testing the Third Thumb design have been limited to exhibition settings (where it is difficult to conduct detailed research), and lab settings (where accessibility to diverse users is relatively restricted; (18)). We wanted to balance these two considerations to ensure that the technology that we are currently studying and iterating can be used by almost anyone, by collecting a large dataset as part of the Royal Society Summer Science Exhibition (Movie 1).

## Results

Our primary goal was to ensure inclusive wearability of the Third Thumb. The Thumb is worn over the ulnar side of the palm, opposite to the user’s natural thumb. Movement of the device is proportionally controlled by a pressure sensor placed under each big toe or foot, to create a dynamic movement with two independent degrees of freedom for the Thumb. Pressure exerted with the right toe pulls the Thumb across the hand (flexion), whereas the pressure exerted with the left toe pulls the Thumb up toward the fingers (adduction); releasing pressure moves the Thumb back to the original position. The extent of Thumb movement is fully proportional to the pressure applied. The adapted design is featured in Figure 1A-B. For inclusive wearability, we developed two sizes of the Thumb: Large and Small. Each have fully flexible, adjustable parts to ensure a snug fit onto the hand for optimal functional use. The rigid wearable hand part, which provides the base for the 3D printed Thumb, is shaped to fit on the side of the hand, an area where there is relatively little fluctuation in size. The adjustable elements are the three straps to secure the Thumb onto the hand and wrist: these play a key role in the size adaptability of the design. Within the context of the exhibition, we simplified the original battery powered and wirelessly controlled Third Thumb design. Two rigid, slim stand-on foot plates were wired directly to the wearable Thumb and wrist unit. This exhibition design decision, as featured in Figure 1C, was made to aid the speed of putting the Thumb onto participants, as there were fewer worn elements, as well as for power and hygiene reasons.

### Examining device wearability in a diverse sample of users

**Figure 2:**
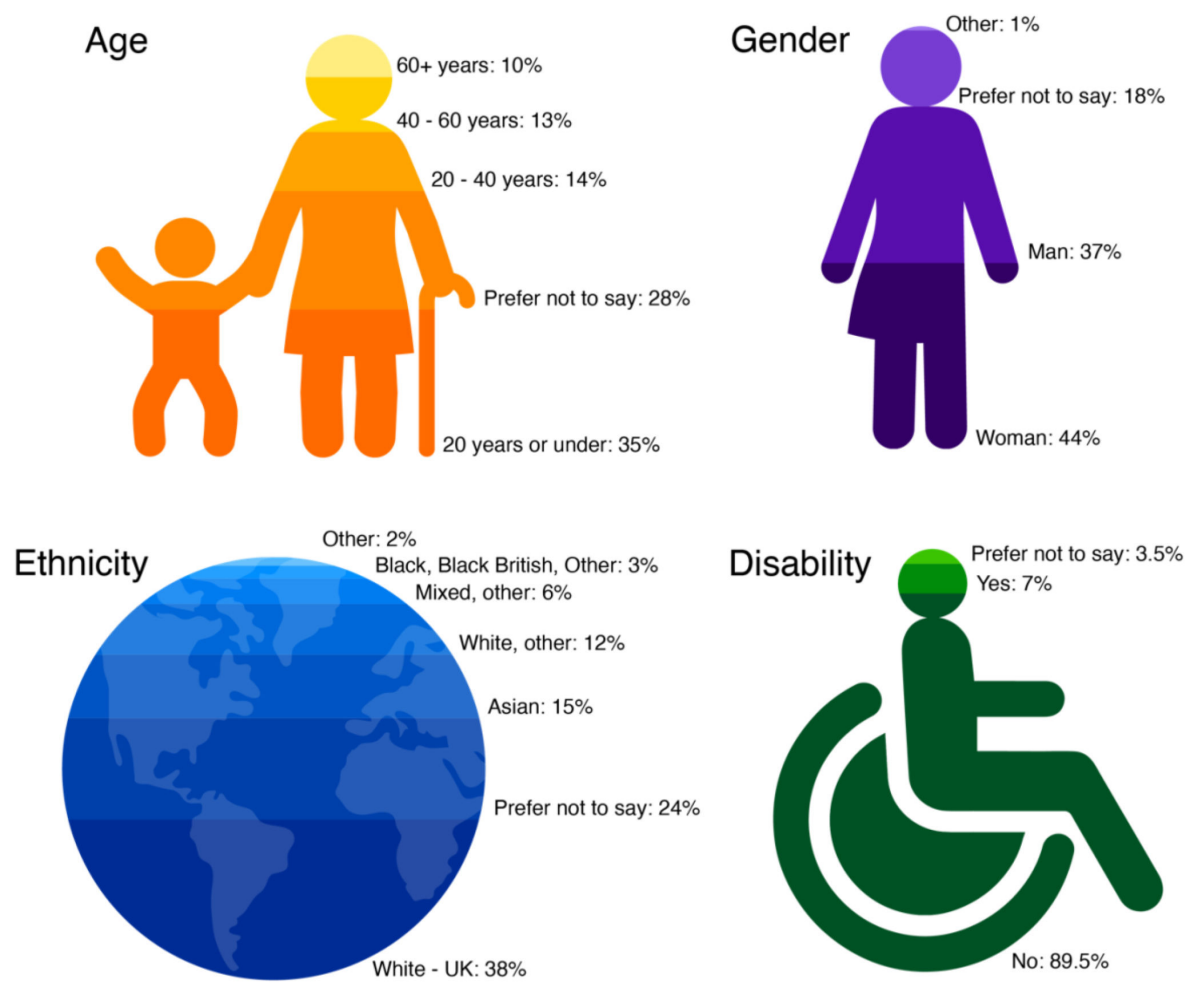
Extended demographics indicators. Demographics taken from a sample of visitors (11%) out of the total 6,774 visitors at The Royal Society Summer Science Exhibition as part of diversity monitoring (19). Please note that these values are only presented for illustration purposes only, as the sample used in this survey (11%) might not have overlapped with the study sample (encompassing 9% of the exhibition visitors).

The Royal Society Summer Science exhibition was visited by over 6,000 visitors, from schools, the media, and the general public (19) (see Figure 2 for cross-sectional diversity monitoring of the exhibition attendants). As part of our exhibit, we successfully tested 596 participants, ranging from ages 3-96 years (see Movie 1 and Table 1)

We specifically focused on wearability (fit and sensor control), ability to successfully operate the device for the first time, and to compare ability-levels across diversity factors that have been previously highlighted as relevant for physical technologies (gender, handedness, and age). This provided us with an extensive opportunity to examine the compatibility of the technology with a diverse group of users.

To test for wearability, the experimenter placed the Third Thumb onto the right hand of each participant. Of the two sizes available, each participant used the Third Thumb that best fit their hand, with the adjustable straps creating a secure fit. To test controllability, we then asked participants to press down onto the left foot sensor and then the right foot sensor that controlled the Third Thumb. This was to ensure the participant could apply enough pressure to appropriately engage the full range of movement of the Third Thumb. The fit and initial ability to move the Third Thumb was then logged by the experimenter.

Of our 596 participants, only 4 were unable to use the Third Thumb, either because the Thumb did not fit their hand securely, or because they were unable to control it with their feet (the pressure sensors were not optimally calibrated for very lightweight children). Therefore, our inclusive design was successfully worn and controlled by 99.3% of our diverse sample.

### Examining successful device performance among a diverse sample of users

Our second goal was to determine that the Third Thumb could easily be used by a highly diverse sample of users. We were particularly interested in the users’ first-time experience because it can influence their overall perception of a novel technology. If a user has a negative experience during their first interaction with a specific technology, they may be less likely to continue using it (20). On the other hand, a positive first-time experience can lead to increased user satisfaction and a desire to continue engaging with it (21).

Importantly, a successful first-time experience can help reduce the learning curve for new users, making it easier for them to become proficient with the technology and take full advantage of its capabilities.

**Figure 3:**
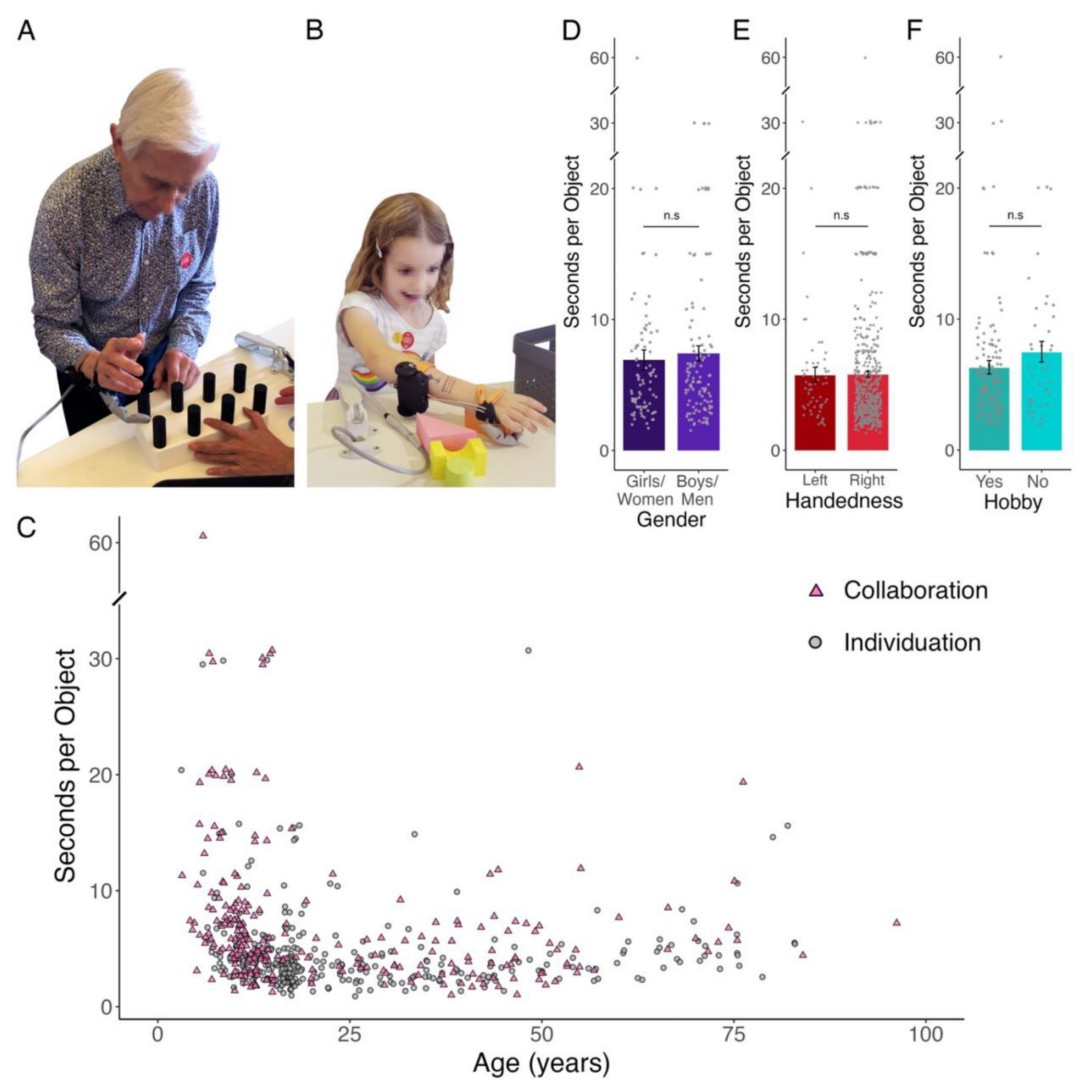
Demographics effects on task performance. Data showcasing the inclusivity of the Third Thumb design. **(A)** A participant completing the individuation task − requiring the user to pick up and transfer individual pegs only using the Thumb (see also Movie S1). **(B)** A participant completing the collaboration task − requiring the user to pick up and transport soft objects using the Thumb in collaboration with their fingers (see also Movie S2); (for full details please see Methods). **(C)** Performance in both tasks (pink triangle for collaboration, grey circle for individuation), based on age (*n* = 578). We find that people of all ages can use the Third Thumb, and there is a general average improvement in performance in adulthood. **(D)** We find no differences in performance across boys/men and girls/women, as demonstrated in the collaboration task, where gender information was collected (error bars denote standard error of the mean, *n* = 196). **(E)** We find no differences in performance across right-handed and left-handed people, demonstrated across the individuation and collaboration task data combined (error bars denote standard error of the mean, *n* = 573). **(F)** We find limited differences in performance across people who have a hobby that involves their hands compared to those who do not (error bars denote standard error of the mean, *n* = 188). BF denotes Bayes Factor (favouring the alternate hypothesis over the null), n.s. denotes non-significant, indicating a BF_10_ ≤ 1, determined by a Bayesian Mann-Whitney test.

We wanted to learn if successful first-time use of the Third Thumb was an option for the broad range of exhibition participants. After confirming they could use the sensors, participants were then given up to one minute to familiarise themselves with the device; during this time, the task was also explained to the participant (see Materials and Methods: Individuation and Materials and Methods: Collaboration below). Participants were then given 60 seconds to complete one of two tasks, involving picking up and transferring as many objects as they could (Figure 3).

The individuation task (Figure 3A and Movie S1) was inspired by grooved pegboard tasks widely used to assess motor functioning and dexterity in clinical neuropsychology (22). A block of eight pegs (Table S1) was placed to the participant’s right and a basket was placed to their left. The participant was instructed to pick up each peg one by one using just the Third Thumb, and transport them into the basket, avoiding engaging their biological fingers in the task. Once all eight pegs have been moved, the experimenter would quickly replace the block with another full block of eight pegs. Participants were asked to move as many pegs as possible in 60 seconds. There were 333 participants who completed this task.

The collaboration task (Figure 3B and Movie S2) involved using the Third Thumb in collaboration with the biological hand to manipulate and move five or six different foam objects. The objects were of various shapes (Table S1) that required different manipulations to be used, increasing the dexterity of the task. The objects were placed to the right of the participant and a basket was placed to their left. The participant was instructed to use the Third Thumb in collaboration with any biological finger (or their palm) to pick up each object one by one and place them into the basket. Participants were then asked to move as many objects as they could into the basket within a maximum of 60 seconds. There were 246 participants who completed this task.

Our extensive testing of people from a wide demographic range allowed us to confirm that almost anyone can use the device straightaway. We found that 98% of participants were able to successfully manipulate objects using the foot-controlled Thumb during the first minute of use, with a median of four seconds per object across tasks (Figure 3C). Of the 13 participants who we considered as unsuccessful, 10 were labelled unsuccessful because they did not incorporate the Thumb while performing the task. This could have been attributed either to an inability to operate the Thumb successfully, or, just as likely − a difficulty to follow the experimenter’s instruction. Since we were unable to dissociate these two causes, in our analysis and interpretation we opted for the more conservative view, where we considered all failed cases to be due to unsuccessful design interface.

### Demographic considerations for augmentation technology ability level

Although 98% of our participants were able to successfully complete our tasks, ability levels between participants were varied. We wanted to understand whether some key demographical factors − which we discussed above as essential for inclusive technological development − are relevant for determining the level of ability to use our hand augmentation technology. As we discuss below, demographic information is not always feasible to collect in the settings of a public exhibition. For this reason, we adjusted our personal questions depending on density of visitors and availability of our exhibition team. After informed consent was obtained, we recorded the date of birth and handedness from all of the participants (n = 596). As reported in Table 1, 10.47% of our participants identified as left-handed − this is highly consistent with the estimated proportion in the UK population (10.4% (23)). For a subset of participants taking part in the collaboration task, we also enquired about their gender (n = 199; of which three participants were considered unsuccessful users). As reported in Table 1, 42.35% of our participants identified as girls/women − this is highly consistent with the estimates from the main exhibition demographics.

We also wanted to explore whether people with jobs or hobbies involving dexterous object manipulation (for example, gaming, knitting) and/or musical experience as a child, could predict people’s ability to use our hand augmentation technology for the very first time (n = 188). These factors were all assessed using a Bayesian Mann-Whitney test with 10,000 samples. We note that task performance did not differ between the group who provided this additional personal information and the group who only provided information of age and handedness (Bayes Factor (BF)_10_=0.20).

Our testing provided us with conclusive evidence that gender does not play a role in technology integration of this kind (BF_10_=0.17). That is, boys/men and girls/women are equally able to use the Thumb (Figure 3D). Similarly, handedness did not change performance (BF_10_=0.14); despite the Thumb being worn always on the right hand, left-handed individuals showed a similar level of ability as right-handers to perform our tasks (Figure 3E). When it comes to people’s use of their hands − either as children (learning to play a musical instrument), in their jobs or for their hobbies − we found anecdotal but not definitive evidence that this was irrelevant to tasks performance (BF_10_=0.35; Figure 3F). In other words, it does not matter if you are a man or a woman, right- or left-handed, or even if you are ‘good’ with your hands − our findings show that everyone can pick up similar levels of ability when using the Thumb for the first time.

### Age considerations for augmentation technology ability level

**Figure 4:**
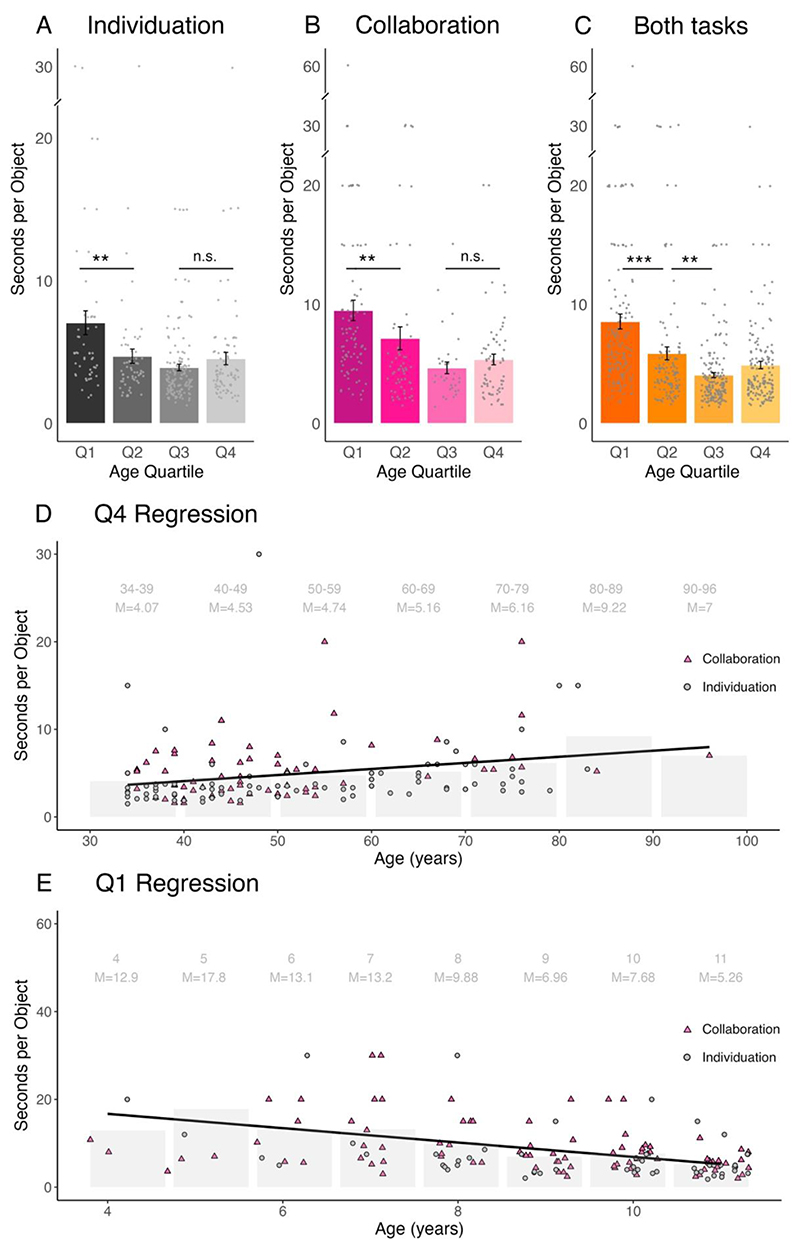
Age effects on task performance. **(A)** For the individuation task, we see older children (12-16 yrs, 2^nd^ quartile, *n* = 63), perform better than younger children (≤ 11 yrs, 1^st^ quartile, *n* = 53). However, there is no difference in performance within adulthood, with younger adults (17-33 yrs, 3^rd^ quartile, *n* = 134) performing similarly to older adults (34-96 yrs, 4^th^ quartile, *n* = 83). Error bars denote standard error of the mean. **(B)** For the collaboration task, we find similar results, with older children (*n* = 59) performing better than younger children (*n* = 87), whilst young adults (*n* = 33) and older adults (*n* = 66) performed at a similar level. Error bars denote standard error of the mean. **(C)** When combining the data across both the collaboration and individuation tasks we begin to see a slightly different pattern. We find older children (*n* = 122) still perform better than younger children (*n* = 140). However, younger adults (*n* = 167) perform better than older children, and older adults (*n* = 149) perform slightly worse than younger adults. Error bars denote standard error of the mean. **(D)** When inspecting only the older adults (34-96 yrs, 4^th^ quartile), we see a linear trend demonstrating degradation of performance with age (BF_10_ = 22.23) **(E)** For the younger child (≤11 yrs, 1^st^ quartile) we see the opposite trend, with a linear improvement in performance with age (BF_10_ = 39.2K). BF denotes Bayes Factor (favouring the alternate hypothesis over the null), ** denotes BF_10_ ≥ 10, *** denotes BF_10_ ≥ 100, n.s. denotes non-significant, indicating a BF_10_ ≤ 1, determined by a Bayesian Mann-Whitney test (A-C) or a permutation linear regression (D-E). M= denotes the mean for each specified age bracket, also shown by the bar plots with standard error of the mean bars. These fluctuations in performance across age raise important questions regarding learning to use motor augmentation technology.

We next considered the effects of age on task performance. We first noticed that older adults (aged 34-96 years, 4^th^ quartile) showed similar abilities to make use of the new technology as younger adults (aged 17-33 years, 3^rd^ quartile). This was demonstrated when comparing the 3^rd^ and 4^th^ quartiles of oldest participants; BF_10_=0.47 and 0.27 for the individuation and collaboration tasks, respectively; Figure 4. However, when the two tasks were put together, we see anecdotal evidence supporting the possibility that older adults performed slightly worse overall than younger adults (BF_10_=2.35). To further explore the influence of ageing on our participants ability to control the Third Thumb, we have examined performance differences within the 4^th^ quartile, which encompasses a relatively very broad age range (34-96 years). We observed that within this age bracket, performance was linearly degraded with age (BF_10_=1.55, 10.27 and 22.23 for individuation, collaboration and combined tasks, respectively; Figure 4D). Therefore, although on average older and younger adults performed the task comparably, we can also identify age costs within the older adults quartile. This effect could be due to the general degradation in sensorimotor and cognitive abilities that are associated with ageing and would influence our task performance. Additionally, these effects could also reflect a generational relationship to technology.

When it came to younger participants, we found some interesting differences which are important to highlight for future research. In particular, six out of the 13 participants that could not complete the task were below the age of 10 years old. Additionally, it was observed that performance was generally poorer among younger children (aged ≤ 11 years, 1^st^ quartile) in comparison to older children (aged 12-16 years, 2^nd^ quartile). This was demonstrated when comparing the 1^st^ and 2^nd^ age-based quartiles of participants (BF_10_=11.75 and 47.39 for individuation and collaboration, respectively; BF_10_=4405.37 when the tasks were combined, Figure 4). To further explore the influence of age on task performance, we again used a permutation linear regression test across children in the youngest age bracket (≤ 11 years), and found a strong linear relationship with age (BF_10_=28.34, 116.57 and 39.2K for individuation, collaboration and combined tasks, respectively; Figured 4E). Opposite to the regression result we found in the older adults, here older children showed improved motor performance with the Third Thumb.

Interestingly, when combining performance across both tasks, even older children (aged 12-16 years) struggled more than young adults (aged 17-33 years; BF_10_=53.44) (Figure 4C). As motor control is already well established in older children, this could be attributed to attention or other cognitive factors which are relevant for motor performance (24). It is important to understand why children and teenagers, who are presumably better emersed in technology, under-performed relative to older users. Are these performance gaps due to motor, attentional, or other cognitive abilities? Would the gap be closed with more training? These are important questions for future research.

## Discussion

### Developing and testing inclusive wearable enhancement technologies

The advancement of motor augmentation and the broader domain of human-machine interaction will ultimately rely upon a seamless integration of users’ motor and cognitive capabilities; in addition to exploring the extent of training necessary for these technologies to be truly beneficial. These considerations may dramatically fluctuate between individuals, based on their form, abilities, and preferences. There is a need to develop a standard by which further research should take these diversity needs and preferences into consideration when guiding technological development.

Although several exoskeleton devices are currently commercially available (for example, ReWalk, Sarcos Robotics), most augmentation devices are still at the early research phase, and generally restricted to lab settings. This presents a unique opportunity to ensure that these technologies are developed inclusively and safely. For wearable technologies specifically, diversity refers to people with a wide range of body types, as well as a range of cognitive and physical abilities. Such varying abilities might relate to age, gender, weight, lifestyle, range of physical and/or cognitive ability and disability, as well as people’s cultural, financial, and societal preferences, for example an affinity, or limited access, to technology. However, for most developers, it is not necessarily easy or straightforward to gain access to information regarding the diversity of their intended consumers. For example, details concerning age, weight, disability, and lifestyle might be confidential and not easily disclosed. Other factors, such as physical and cognitive ability, require customised testing which is not immediately available for many developers. Cultural and societal preferences might change from one geographical location to another, making these preferences difficult to access. In addition, these different factors might associate with each other in complicated ways (25), meaning that addressing diversity requires very large samples.

We believe that physical testing of large and diverse groups of individuals is essential to achieve this goal. Yet, the empirical output around these core diversity considerations is extremely limited in the realm of augmentation technologies. Most published studies investigating sensorimotor control of extra robotic body parts are based on highly narrow samples, ranging from one single participant in some (for example, (26)) to, at most, 44 participants (18) (median sample, based on a recent review = 10; (3)). Even in the largest sample to date, the age range was limited to 18-35 years. This reflects a need to test the wearability, functionality, and acceptance of these augmentation technologies on a broader range of potential users. To study the wearability and first-use experience, we collaborated with the Royal Society Summer Science Exhibition in London, where our augmentation device the Third Thumb was exhibited as an interactive display over five days. This allowed us to reach a diverse population of a wide demographic that may not have been otherwise included in the research and development of a novel wearable technology.

### Study limitations

We were particularly fascinated with users’ first-time experience because it can influence their overall perception of − and motivation towards adopting − a new technology. Our implicit assumption was that if participants don’t have a successful first-time experience then this will create barriers for inclusion and user diversity. As such, our explicit aim was to test participants from the general public that are often not involved in early development of wearable robotics technology, with a particular focus on gender over a broad range of age, and handedness. In this context it is important to highlight that, although we did not identify significant barriers for specific participant demographics, it is very possible that more differences will emerge with more training. Indeed, the tasks used here were tailored for using a hand augmentation device with minimal training. However, the complex process of skill learning, which is comprised of both sensorimotor and cognitive abilities (27), might provide users, for example, of different age brackets, different opportunities to develop high level of dexterity to use the device. As such, future research will be needed to examine diversity considerations in the context of long-term practice and habitual use.

Public engagement events provide an important opportunity to develop a bi-directional discourse for the co-development of inclusive and accessible technologies; and large-scale testing can provide us with conclusive evidence to inform technological development. However, our ambition to collect data from as many exhibition attendees as possible highlighted certain constraints. Although our focus remained on maximising participant numbers, particularly first-time users, this often came at the expense of deeper interactions and comprehensive user profiling. Additionally, given the public setting of our interactions, safeguarding participant privacy was a core concern. This meant that we were limited to collecting only a small sample of diversity factors, and in a limited number of participants. Although the efforts of the exhibition organisers provide us with more comprehensive indications of the makeup of the exhibition visitors, these should be taken with caution: with the exception of gender and handedness, we don’t have evidence to indicate that our sub-sample of users reflected the multiple axes of diversity which are relevant for technological inclusion. In particular we were unable to provide a comprehensive cross-sectional characterisation of many other important diversity considerations, such as ethnicity and disability. Those will need to be investigated in future research.

### Concluding remarks

Through our work, we have offered a tangible demonstration of the initial usability of the Third Thumb device for a broad demographic, and additionally hope that our approach, as well as our promising results, may pave the way for establishing a benchmark for inclusive technological development-stage testing for other human centred technologies.

## Materials and Methods

We aimed to collect first-exposure data from members of the general public using the Third Thumb for the first time at the Royal Society Summer Science Exhibition 2022 (*n* = 596). Following wearability checks, a diverse range of participants carried out one of two, one-minute tasks aimed at different motor skills (individuation or collaboration) with the Third Thumb. Demographic information and simple performance measures were obtained for each participant and detailed below.

### Participants

The study was ethically approved by the UCL Research Ethics Committee (Project ID number:12921/001 and 17205/001). We collected data from 600 participants, although four participants withdrew their participation during the session. Of the remaining 596, one participant (aged three) did not fit the Third Thumb. A further three participants (aged five,six and seven) were not able to apply enough pressure to engage the sensor. This was because the sensors were not optimally calibrated for the weights of very small children.

Out of the 592 participants remaining, three participants were not able to complete the task, and a further 10 participants did not engage the Third Thumb in the task as instructed and their performance was thus considered a fail. This meant out of 592 participants, 97.8% of participants successfully used the Third Thumb to engage in an object manipulation task within a minute of using it. Six out of the 13 unsuccessful participants were under the age of 10. Table 1 shows the demographic information for the 579 successful participants.

### The Third Thumb

The Third Thumb is a robotic supernumerary finger that is worn on the ulnar side of the right hand. The Third Thumb is normally worn on the hand and wrist, and powered by a battery pack that sits on the bicep, it is then controlled wirelessly by pressure sensors secured underneath the big toes (full description in (17)). In our main device model, the coin -sized toe-controlled pressure sensors are worn inside the shoe. However, given the demands of the exhibition (an intense week of continuous usage, with a large turnover of people using the Third Thumb, each for a short period), the design was adapted. Here the Third Thumb was powered directly from a mains power supply - so battery packs did not need to be constantly recharged. The device was instead controlled via two external foot pads with imbedded pressure sensors, which were placed on the ground in front of the relevant Third Thumb on the exhibition table, to service easy access for the user, who could leave their shoes on. With two degrees of freedom, applying pressure to the sensor on the left caused a proportional adduction/abduction movement, applying pressure to the sensor on the right caused a proportional flexion/extension movement. We note that this adaptation provides a very similar solution to the fully wearable Thumb, with the main difference being the pressure sensors are outside the shoe, rather than inside, and that we were not able to customise the pressure sensors to individual’s weight and personal preferences.

The adapted exhibition model only required the main Thumb and wrist elements to be worn by the participant, and this was achieved within a few seconds. Four Third Thumbs were available in two different sizes - two small Third Thumbs intended to fit the hand of a younger child and adults with small hands, and two large Third Thumbs intended to the fit the hand of the average adult. All participants used the Third Thumb that best fit their hand, ensuring people of varied hand sizes could participate. The adjustable straps of the Third Thumb insured a secure fit.

### General Procedures

The study was conducted at the Royal Society Summer Science Exhibition 2022. The exhibition was hosted at The Royal Society in London, England, and lasted five days (6^th^ July − 10^th^ July). Upon entering the exhibition, the Royal Society obtained permission to photograph and video attendees. Before taking any photos or videos, we also obtained verbal consent from the participant and their guardian when the participant was under 18.

Anonymous study data is shared at 10.5281/zenodo.11005439. After informed consent was obtained, demographics information about date of birth and handedness was collected (*n* = 596), as well as gender (*n* = 199), if they had jobs or hobbies involving use of the hands (for example, gaming, knitting) and musical experience as a child for later participants when feasible (*n* = 188). The experimenter then fitted the Third Thumb onto the right hand of the participant and asked them to press down onto the left foot sensor and then the right foot sensor that controlled the Third Thumb. This was to ensure the participant could apply enough pressure to appropriately engage the pressure sensors to produce Third Thumb movement. The fit and ability to move the Third Thumb was logged by the experimenter. Participants were then given up to one minute to familiarise themselves with the device, during this time the task was also explained to the participant (see Materials and Methods: Individuation and Materials and Methods: Collaboration below). Participants were then given 60 seconds to complete the full task (see outcome measures in Materials and Methods: Individuation and Materials and Methods: Collaboration).

Participants visiting on the first three days of the exhibition completed the individuation task, participants visiting on the last two days completed the collaboration task.

### Individuation task

The individuation task was inspired by grooved pegboard tasks widely used to assess motor functioning and dexterity in clinical neuropsychology (22). A block of eight pegs (Table S1) was placed to the participant’s right and a basket was placed to their left. The participant was instructed to pick up each peg one by one using just the Third Thumb, move and place them into the basket, not engaging their biological fingers in the task (Movie S1). The flexion movement allowed the participant to conform the flexible Thumb around the peg and secure it to the palm, whereas the abduction movement allowed the participant to adjust the position of the Thumb relative to the peg to stabilise it. In the familiarisation phase, participants were asked to try to move one peg. Participants were then asked to move as many pegs as possible in 60 seconds. Once all eight pegs have been moved, the experimenter would quickly replace the block with another full block of eight pegs. The experimenter would time for 60 seconds using a stopwatch; after 60 seconds had passed, the number of counted pegs moved were recorded.

### Collaboration task

The collaboration task involved using the Third Thumb in collaboration with the biological hand to manipulate and move five or six different foam objects (Table S1). The objects were of various shapes that required different manipulations to be used, increasing the dexterity of the task. The objects were placed to the right of the participant and a basket was placed to their left. The participant was instructed to use the Third Thumb in collaboration with any biological finger (or their palm) to pick up each object one by one and place them into the basket (Movie S2). In the familiarisation phase the participant was given a square object with a large hole in its middle to practice moving, as it was the easier object to grasp. Participants were then asked to move as many objects as they could into the basket whilst the experimenter recorded the time taken using a stopwatch, with a maximum of 60 seconds.

### Statistical Analysis

All analyses were run in R version 4.2.2 (R Core Team, 2022) and JASP version 0.17 (JASP Team, 2023).

For data analysis, an outcome measure of seconds per object was calculated for both tasks. For the individuation task, this was the number of pegs moved divided by 60. For the collaboration task, this was the number of objects moved divided by the time taken (in seconds) to move them (up to 60).

Analyses used non-parametric statistics to ensure the results were not inflated by the presence of extreme values and unequal groups sizes. All results are reported using Bayes Factors (BF_10_), a statistical measure that provides evidence in favour of one hypothesis over another based on the data observed. The BF_10_ represents the BF in favour of the alternative hypothesis over the null hypothesis. All interpretation is based on well-established criterion (28). We used a Cauchy prior width set to 0.707 throughout. All BF_10_ values were obtained using 10,000 samples. Full statistical results, including p-value and effect size, for each result reported can be found at 10.5281/zenodo.11005439.

To investigate the effect of age on performance, we first ran a Mann-Whitney test to check if the two task groups differed in age of the sample. We found they did differ (BF_10_= 97.67), so we then proceeded to complete the analysis twice − once for the individuation task, then for the collaboration task. We first treated age as a continuous variable and ran a linear permutation test. We then split the whole sample into four age groups, based on quartiles to ensure there was a roughly equivalent number of participants in each group. This meant the younger children made up the first quartile (≤11, *n* = 140), older children made up the second quartile (12-16, *n* = 122), young adults made up the third quartile (17-33, *n* = 167), and the fourth quartile was made up of older adults (34-96, *n* = 149). To investigate the main effect of quartile we used a Kruskal-Wallis test, followed up with a Mann-Whitney test to compare specific quartiles of interest. We compared the quartiles within the individuation task, within the collaboration task, and then with the data from both tasks combined.

To explore the effect of handedness on performance, we first removed ambidextrous participants from the analysis due to having such a small sample (*n*=5). We then used a Chi-Square test to see if the two task groups differed in handedness and found no difference between groups (BF_10_ = 0.205). Therefore, we used a Mann-Whitney test to explore the effect of handedness across both task groups (left-handed *n* = 60, right-handed *n* = 513).

To see if gender affected performance on the collaboration task, we used a Mann-Whitney test comparing performance between boys/men (*n* = 113) and girls/women (*n* = 83). Note that we did not collect information about gender identity from the full sample.

For a subset of participants that carried out the collaboration task, we also asked whether they had a job or a hobby that involved use of their hands, or if they played an instrument as a child. To avoid confusion with younger participants, most children were only asked if they played an instrument. This meant we had a total of 92 responses for the job and hobby question, and 187 responses for the instrument question. We wanted to explore if experience using their hands for dexterous tasks influenced Third Thumb performance. Therefore, we combined these two variables to create a new hobby variable. For this combined variable, 149 people had a hobby involving their hands and/or played an instrument as a child, whilst 39 did not. We compared these groups using a Mann-Whitney test.

## Supplementary Material

Supplementary Material

Supplementary Table

## Figures and Tables

**Table 1:** Demographics Summary. Demographics data for all participants that successfully performed the task, split by task group. For Handedness, L = left; R = right; A = ambidextrous. Please note that diversity information was not fully sampled.

Task	N	Age (years)	Handedness	Gender	Performance (per object)
Individuation	333	*M* = 25.76 (4-83)	L = 31, R = 299, A = 3	N/A	Median = 3.5 seconds
Collaboration	246	*M* = 23.29 (4-96)	L = 29, R = 214, A = 2	Boys/Men = 113 Girls/Women = 83	Median = 5.3 seconds

## Data Availability

All data and analysis scripts needed to evaluate the conclusions in the paper are available at 10.5281/zenodo.11005439. This research was funded in whole or in part by the European Research Council (715022 Embodied Tech), Wellcome Trust (215575/Z/19/Z) and Medical Research Council (MC_UU_00030/10), cOAlition S organizations. The author will make the Author Accepted Manuscript (AAM) version available under a CC BY public copyright license.

## SUMMARY

Most participants successfully used the Third Thumb within one minute - demonstrating usability of design by diverse users.
