# Supplementary material for: Evaluating initial usability of a hand augmentation device across a large and diverse sample
Source: Sci Robot. Author manuscript; Available in PMC 2024 Jul 29. (PMC7616312; doi:10.1126/scirobotics.adk5183)
Supplement: Supplementary Material [file EMS196572-supplement-Supplementary_Material.zip › scirobotics.adk5183_sm.pdf]

Science Robotics
AAAS

Supplementary Materials for

Evaluating initial usability of a hand augmentation device across a large and diverse sample

Dani Clode *et al.*

Corresponding author: Tamar R. Makin, tamar.makin@mrc-cbu.cam.ac.uk

Sci. Robot. **9**, eadk5183 (2024)
DOI: 10.1126/scirobotics.adk5183

The PDF file includes:

Table S1

Other Supplementary Material for this manuscript includes the following:

Movies S1 and S2
MDAR Reproducibility Checklist

Table S1: Task Equipment Details. Dimensions and weight of the pegs used in the individuation task and the foam objects used in the collaboration task.

Name	Dimensions (mm)	Weight (g)	Image
Adult's peg	84x23mm (dia)	20g	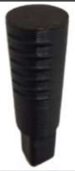
Children's peg	69x19mm (dia)	13g	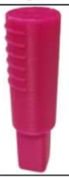
Foam triangle	69x69x96x35mm	8g	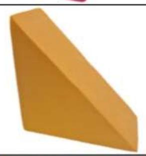
Foam bridge	34x70x34mm	6g	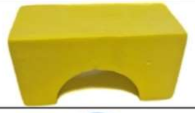
Foam square	69x69x34mm	8g	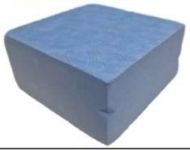
Foam square with hole	69x69x34mm	13g	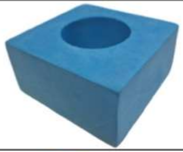
Medium foam rectangle	34x45x69mm	4g	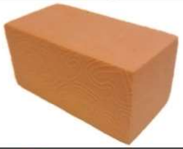
Long foam rectangle	34x34x139mm	15g	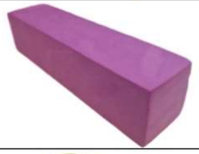
Small foam cylinder	34x34mm (dia)	2g	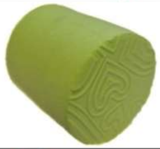
Medium foam cylinder	70x34mm (dia)	3g	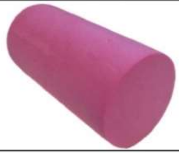
