# Supplementary material for: Evaluating initial usability of a hand augmentation device across a large and diverse sample
Source: Sci Robot. Author manuscript; Available in PMC 2024 Jul 29. (PMC7616312; doi:10.1126/scirobotics.adk5183)
Supplement: Supplementary Table [file EMS196572-supplement-Supplementary_Table.pdf]

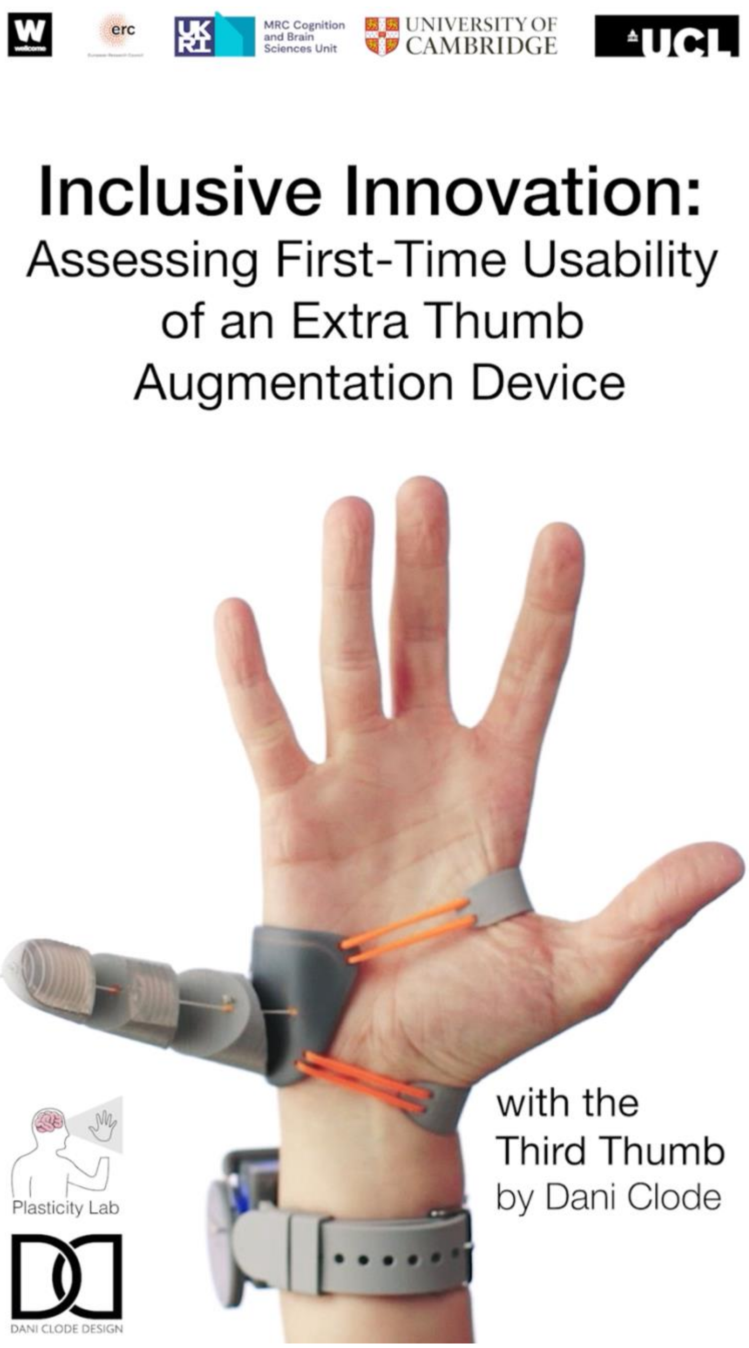

Movie 1: Third Thumb at the Royal Society Summer Science Exhibition 2022.

Supplementary Materials and Methods

Table S1
Movies S1-S2

References:

SUPPLEMENTARY MATERIALS

TITLE

Evaluating initial usability of a hand augmentation device across a large and diverse sample

SHORT TITLE

First-time Usability of an Extra Thumb

AUTHORS

Dani Clode[1,2,3*], Lucy Dowdall[1,2,*], Edmund da Silva[1,2], Klara Selen[1], Dorothy Cowie[4], Giulia Dominijanni[1,5], Tamar R. Makin[1,2]

[1] MRC Cognition and Brain Sciences Unit, University of Cambridge, Cambridge, UK
[2] Institute of Cognitive Neuroscience, University College London, London, UK
[3] Dani Clode Design, Cambridge, UK
[4] Department of Psychology, Durham University, Durham, UK
[5] Bertarelli Foundation Chair in Translational Neural Engineering, Neuro-X Institute, Ecole Polytechnique Fédérale de Lausanne, Lausanne, Switzerland
* These authors contributed equally to the study.

Table S1: Task Equipment Details. Dimensions and weight of the pegs used in the individuation task and the foam objects used in the collaboration task.

Name	Dimensions (mm)	Weight (g)	Image
Adult's peg	84x23mm (dia)	20g	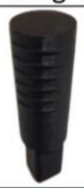
Children's peg	69x19mm (dia)	13g	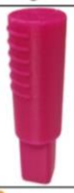
Foam triangle	69x69x96x35mm	8g	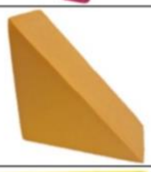
Foam bridge	34x70x34mm	6g	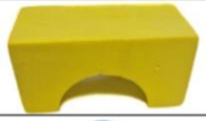
Foam square	69x69x34mm	8g	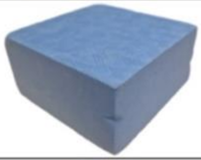
Foam square with hole	69x69x34mm	13g	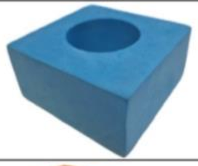
Medium foam rectangle	34x45x69mm	4g	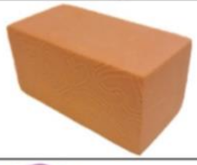
Long foam rectangle	34x34x139mm	15g	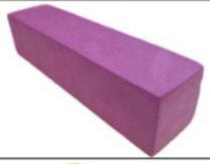
Small foam cylinder	34x34mm (dia)	2g	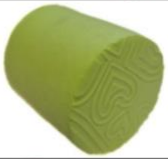
Medium foam cylinder	70x34mm (dia)	3g	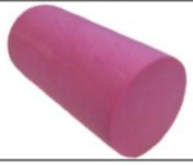
